# To What Extent Does Clinically Assisted Nutrition and Hydration Have a Role in the Care of Dying People?

**DOI:** 10.1177/0825859720907426

**Published:** 2020-03-04

**Authors:** Adam Nicholas Carter

**Affiliations:** 1 Merton College, 61483 University of Oxford, Oxford, United Kingdom

**Keywords:** clinically assisted nutrition and hydration, hospice care, hydration, medical ethics, nutrition, palliative care

## Abstract

The question over whether to administer clinically assisted nutrition and hydration (CANH) to a dying patient is controversial, with much debate concerning this sensitive issue. The administration of CANH poses clinical and ethical dilemmas, with supporting and opposing views. Proposed positive effects of CANH include preventing thirst, delirium, hypercalcemia, and opioid toxicity. However, CANH has been shown to increase the risk of aspiration, pressure ulcers, infections, and hospital admissions as well as potentially causing discomfort to the patient. Guidance from several national bodies generally advises that the risks and burdens of CANH outweigh the benefits in the dying patient. However, an individualized approach is needed, and the patient’s wishes regarding CANH need consideration if they have capacity and can communicate. Otherwise, sensitive discussions are required with the family, enquiring about the patient’s prior wishes if there is no advanced care plan and acting in the patient’s best interests. The ethical principles of autonomy, beneficence, non-maleficence, and justice need to be applied being mindful of any cultural and religious beliefs and potential misperceptions.

## Introduction

Food and drink are basic physiological needs, with psychological, social, and symbolic significance.^1^ In the last days or hours of life, patients gradually become less able, or refuse, to eat or drink by mouth. They should be supported to eat and drink safely for as long as they wish as part of basic care. However, the question of whether to provide clinically assisted nutrition and hydration (CANH), defined in law as medical treatment,^2,3^ has long been debated.

Clinically assisted nutrition and hydration can be divided into clinically assisted nutrition (CAN) and clinically assisted hydration (CAH). It includes intravenous parenteral nutrition and intravenous hydration, nasogastric tube (NGT) feeding, and the placement of surgical feeding devices, including percutaneous endoscopic gastrostomy (PEG), percutaneous endoscopic jejunostomy, and radiologically inserted gastrostomy. Due to the recent tightening of terminology, the phrase “end of life” now refers to patients likely to die within the next 12 months, and the term “dying” to patients in the last days or hours of life.^4^

Prospective trials on CANH are not feasible or ethical in care of the dying. Health-care professionals need to make decisions with the patient and family at a time of high emotion.^5,6^ As medical treatments, the initiation, termination, and withholding of CANH need to be medically and ethically justified.^7^

This essay will review the mechanism of different types of CANH, relevant laws, guidance, and ethical considerations. It will discuss cultural and religious differences, perceptions, and training needs, followed by a discussion.

## Mechanism

In terms of considering the use of CANH in the dying patient, it is important to try to identify when a patient’s body is starting to shut down because of disease and the dying process. Anorexia and cachexia tend to ensue; at this point, nutritional support is normally not beneficial, since nutrients are no longer metabolized as before,^8^ and patients generally do not experience hunger or thirst. Patients, especially those with cortical degeneration, are often unable to eat due to dysphagia.^9^ Nevertheless, loss of appetite and reduced oral consumption in palliative care can sometimes be due to reversible causes, which should be addressed if possible; these include medication side effects, oxygen therapy, constipation, mouth breathing, nausea, pain, anxiety, and depression.^10,11^

Proposed positive effects of CANH include preventing thirst, delirium, hypercalcemia, and opioid toxicity through increasing renal perfusion.^12^ Negative effects of CANH include peripheral edema and increasing cardiac failure due to fluid overload and worsening of vomiting, diarrhea, bloating, cramps, and respiratory secretions.^13^ Another disadvantage of CANH is that ketones and opioid peptides produced in dehydration and malnutrition may have sedative and analgesic effects.^14^

**Figure 1.**
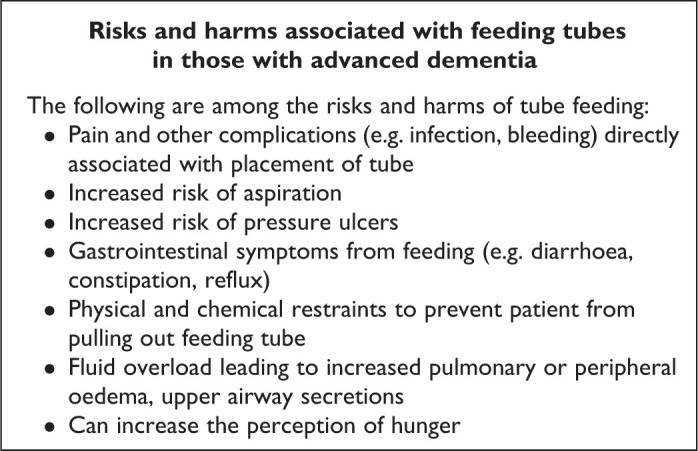
Risks and harms associated with feeding tubes in those with advanced dementia. Adapted from Ying.^17^

There is evidence that CANH often does not benefit patients with advanced dementia^15^ or improve nutritional status.^16^ Moreover, it has recognized risks and harms (Figure 1).^17^ Studies have shown that tube feeding causes an increased risk of aspiration (due to disordered esophageal peristalsis and reflux of the liquid feed), pressure ulcers (due to diarrhea), infections, and hospital admission.^18-21^ These factors are likely to worsen quality of life and shorten life expectancy for patients. Moreover, patients may not tolerate tubes well due to discomfort, leading to restraint to prevent pulling them out.^22^

Local complications of PEG include bowel obstruction, perforation, or tube dislodgement with no insignificant procedure-related mortality rates of 1% to 2%.^23^ Tube feeding also has the negative effects of losing the taste and texture of food and the social and human contact that come with being hand fed. Also, it is felt that it is dryness of the oral cavity rather than pure thirst that causes patient discomfort at the end of life; this can be addressed by lip moisturizing and mouthwash.^24^

## Law and Guidance

The National Health Service (NHS) Long Term Plan emphasizes the importance of care that is “more differentiated,” recognizing that the NHS needs a fundamental shift toward “more person-centered care.”^25^

The Liverpool Care Pathway (LCP) for the Care of the Dying Patient^26^ was the guidance used from the late 1990s until 2014. It was the key policy to improve end-of-life standards, but strict adherence led to nonindividualized care, and there were reports of CANH being withdrawn without explanation or consultation. An independent review “More Care, Less Pathway”^27^ recommended individualized end-of-life care plans, backed up by condition-specific good practice guidance.

**Figure 2.**
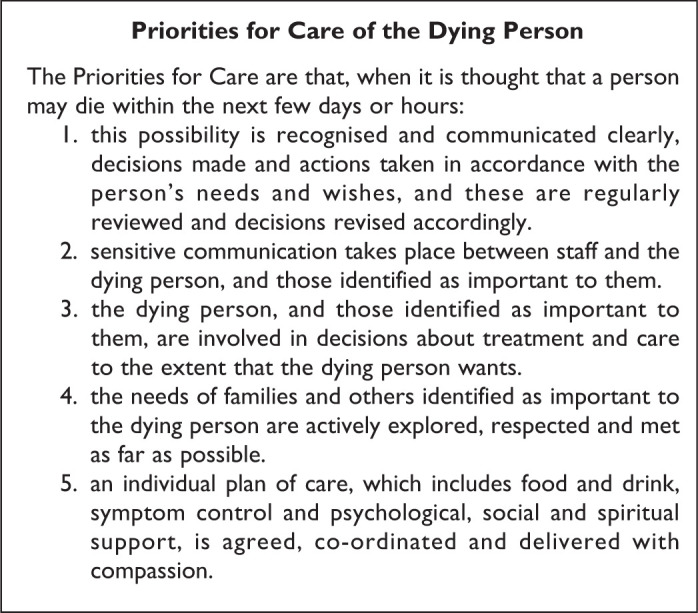
Priorities for Care of the Dying Person. Adapted from: Leadership Alliance for the Care of Dying People.^4^

As a result, the Leadership Alliance for the Care of Dying People (LACDP) published One Chance to Get it Right (OCTGIR), a report setting out a new approach to the care of dying people in England.^4^ It advises that patients should be offered food and drink by mouth if safe to do so and identifies 5 priorities for care as the new basis for caring for a dying patient (Figure 2).

With respect to CANH, Oral Feeding Difficulties and Dilemmas,^28^ published by The Royal College of Physicians (RCP), recommends early discussions regarding preferences for end-of-life care in patients with progressive conditions and good mouth care when oral intake is no longer possible. They advise that for the dying patient, “discontinuation of intravenous fluids must be considered, as it often only serves to exacerbate pulmonary edema, peripheral edema, and increased secretions.” They also advise reviewing the appropriateness of continuing PEG or NGT feeding, with clear reasons identified for withdrawal of CANH, rather than blind adherence to a protocol.

Guidance by the General Medical Council (GMC)^29^ for the care of adults expected to die in hours or days states that if the “burdens or risks of providing CANH outweigh the benefits they are likely to bring, it will not usually be appropriate to start or continue treatment.” They highlight that when benefits, burdens, and risks are finely balanced, the patient’s wishes will usually be the deciding factor.

The National Institute for Health and Care Excellence (NICE) guidelines^30^ for the care of dying adults in the last days of life regarding hydration are “to support the dying person to drink if they wish to and are able to, and consider a therapeutic trial of CAH if the person has distressing symptoms or signs that could be associated with dehydration, such as thirst or delirium, and oral hydration is inadequate.” They advise regular review, employing an individualized approach, considering previous wishes, and any known beliefs, advanced statement, or advanced decision to refuse treatment (ADRT).

For patients on CANH in a permanent vegetative state or minimally conscious state, prior direction had been to seek court approval before withdrawing CANH, based on a combination of case law,^3^ the Court of Protection’s Practice Direction 24B,^31^ and the Mental Capacity Act 2005 (MCA) Code of Practice.^32^ However, on July 30, 2018, the Supreme Court gave judgment on the case of Mr Y, confirming that it is no longer necessary to seek approval from the court for the withdrawal of CANH, providing that the MCA is being followed, relevant guidance is adhered to and that family and health-care professionals agree as to the best interests of the patient.^2^ If there is disagreement, an application to the Court of Protection may be still made. While many welcomed the clarity provided by the judgment, there has been opposition. Professor Charles Foster argues that the judgment risks “making doctors the sole de facto decision makers” and worries about the “algorithmic formulation of guidelines.”^33^

The MCA^34^ was introduced in England and Wales to give a framework to assess the capacity of individuals to make decisions for themselves. The Act is underpinned by 5 statutory principles (Supplemental Figure S1). There is a 2-stage test to assess capacity (Supplemental Figure S2).^35^

**Figure 3.**
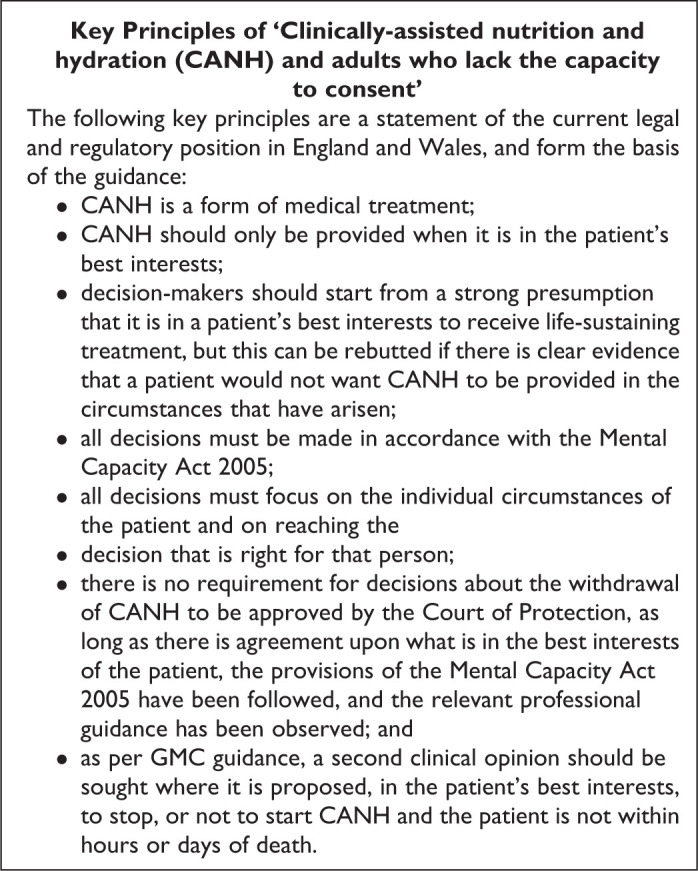
Key Principles of “Clinically-assisted nutrition and hydration (CANH) and adults who lack the capacity to consent.” Adapted from: Royal College of Physicians, British Medical Association.^36^

In December 2018, the British Medical Association (BMA) and RCP jointly published new guidance for making decisions to stop, start, or continue CANH for adults without capacity.^36^ The following key principles represent the current laws and regulations in England and Wales (Figure 3).^36^

They advise withholding CANH where it would provide risks or no clinical benefit, such as in patients with end-stage dementia where it is not expected to prolong life.^37^ When patients are expected to die within hours or days, the clinical reasons against CANH should be sensitively explained to the patient and/or relatives. If CANH is administered, clear goals and regular reevaluations are necessary.^38^

## Ethics

Since 1992, it has been legally established that CANH is a form of medical treatment, rather than part of basic care.^3^ However, there is an emotional and ethical significance attached to CANH that singles it out from other forms of life-sustaining treatment.

In 1989, bioethicist Dr Mark Yarborough questioned the growing use of tube feeding, comparing it to “force-feeding” that may provide the body with more nutrients than it can tolerate.^12^

The European Society for Clinical Nutrition and Metabolism guidelines on ethical aspects of artificial nutrition and hydration were developed by an international multidisciplinary working group in 2016. Their guidelines for the prerequisites of artificial nutrition and hydration are (1) an indication for medical treatment; (2) the definition of a therapeutic goal to be achieved; and (3) the will of the patient and his or her informed consent. They state that, in all cases, the treating physician has to take the final decision and responsibility.^11^ They highlighted that the 4 ethical principles of autonomy, beneficence, non-maleficence, and justice need to be applied during decisions about CANH.^39^

The principle of *autonomy* means considering the patient’s wishes regarding any treatment. If a patient has previously asked for CANH to be provided until death, or family feel that this is what the patient wanted, then these wishes should be accounted for when weighing up the risks and benefits.^40^ The patient’s request will usually be the deciding factor if the balance is close. However, if after discussion it is considered that the treatment would not be clinically appropriate, it does not need to be provided. Palliation is not a withdrawal of treatment but a reprioritization to respect autonomy, give comfort, relieve distress, and reduce treatment burden.^41^ In this circumstance, an explanation should be given to the patient or relatives, discussing other options including seeking a second opinion or the recommendations of a clinical ethics committee. Voluntary cessation of CANH is a legal and acceptable decision of a competent patient but should not be confused with depression or loss of appetite due to disease. A patient’s wishes may change in the dying phase.

To satisfy the principles of *beneficence* and *non-maleficence*, CANH must benefit and not harm the patient; it should not extend the dying phase. If a patient is expected to die within hours or days, CANH is not indicated if the burdens or risks outweigh potential benefits. If the case is borderline, a trial of CANH may be given, with regular reviews of the patient’s condition. If CANH is to be stopped, this decision needs to be communicated to the patient or representatives. Best quality end-of-life comfort care should always be provided.

*Justice* involves distributing resources fairly and without discrimination. For patients with chronic diseases, CANH can be effective until the dying phase, but with increasing age and comorbidity come increasingly difficult ethical decisions.^42,43^ Patients should have the best care possible but if CANH only prolongs the death, it is not justified. Continuing careful feeding by mouth is probably appropriate in many cases. Staff may worry about the risk of aspiration and feel that the patient should be “nil by mouth,”^44^ but the enjoyment of food and social interaction are likely to weigh in the patient’s best interest. Health-care workers need to be able to balance compassionate care with ethical professional standards.^45^

## Cultural and Religious Differences

Patients and their families often find that religion helps them to develop positivity and integrity to help them cope with illness.^46^ Staff need to be respectful of a patient’s culture and religion. They should be mindful of the possibility of family coercion, considering the fact that a patient’s views may oppose those of their family. However, the bonds between the patient and their family are often very close in these emotional final days, so doctors need to be sensitive in these discussions so as not to cause upset. Staff also need to ensure that their own beliefs do not bias any discussions.

In Eastern culture, it is common for the dying patient not to be informed of their prognosis on the basis of non-maleficence, leaving palliative care decisions to their family. A Chinese study, limited by possible bias from being carried out solely in a tertiary center and from some insufficient data due to its retrospective design, found that 97.2% of end-of-life decisions were made between the doctor and the patients’ families.^47^ Conversely, in Western culture, patient autonomy is the primary determinant in end-of-life decisions.^48^ However, this is a complex area: The norms on which decisions are made shift over time, regardless of place or culture.

In 1957, Pope Pius XII declared that life-prolonging treatment such as CANH was extraordinary and idolatry. He felt that care of the dying should focus instead on reducing suffering.^49^ Conversely, Pope John Paul II saw no distinction between CANH and non-CANH, stating that the administration of food and water “always represents a natural means of preserving life, not a medical act,” referring to the withdrawal of nutrition as “true and proper euthanasia by omission.”^50^ Protestant Christianity generally has a more liberal viewpoint that CANH can be used if beneficial but not to prolong life without quality.^11^

In Islam, food is a basic right and not a treatment; therefore, starvation is considered worse than the complications of CANH. However, CANH can be withheld or withdrawn from a terminally ill Muslim patient with informed consent from the patient, family, health-care providers, and religious scholars.^51^

In Hinduism, the cultural belief is that a person reduces oral intake to prepare for a dignified death, and reduced food consumption is a sign of death and not a cause.^52^

Under Jewish law, life should be preserved, so CANH should not be withdrawn if it has been a continuous treatment, and withholding CANH is prohibited and considered to be euthanasia.^53^ However, if it is known that the patient does not want CANH, it may be withheld.^54^

In Buddhism, CANH is supported by some, since it is felt that the patient’s soul will be restless if they die hungry. On the other hand, excessive CANH is detrimental to enlightenment and inspiration which help in the afterlife.^52^

## Patient, Family, and Media Perceptions

Many patients refuse CANH if it will not cure them.^52^ However, despite guidelines generally erring against CANH in the last days and hours of life, in several studies, the majority of patients and families were in favor of CANH,^55^ many feeling that CANH reduces dehydration and pain and prolongs life.^56^ Families may feel that by pushing for CANH, they are benefiting their loved one. Media portrayal of “starvation to death by the NHS” or “back-door euthanasia” may further fuel feelings that care is inferior if CANH is not offered.^57-59^

Unsuccessful attempts to increase the body weight of patients is a major cause of psychological burden for families. Indeed, there is evidence to suggest that weight loss and loss of appetite could be more distressing for the family than the patient.^60^ Holden suggests that this burden could be more pronounced in female relatives.^61^ Pressure put on patients to eat to satisfy their family can lead to distress and feelings of failure in the patient. Rather than taking away what little control patients have in the palliative care setting, it may be best to allow patients the freedom to eat if they wish.^62^ Amano et al suggest that eating-related distress may be alleviated by sufficient explanation about the reasons for anorexia and weight loss in dying patients.^63^

McClement et al found a marked variability in the responses of family members to a dying patient with anorexia and cachexia.^64^ Undertaking interviews with patients and their families, they identified 3 common approaches by families: “fighting back,” “letting nature take its course,” and uncertainty (described as “waffling”). The “fighting back” describes family members who pushed for CANH, fearing patients would otherwise “starve to death.” The “letting nature take its course” group describes family members who focused on other nurturing activities aside from nutritional care. Family members described by the “waffling” group were uncertain about what was best, appreciating that declining food intake was both inevitable and something that they wished to prevent.

Education by doctors, nurses, and dieticians is key to helping patients and families understand about weight loss associated with anorexia and cancer cachexia and in so doing reduce their distress about eating and CANH.^63^

## Staff Training

Health-care professionals need consistent training in CANH. Despite the limited evidence that CANH improves the health of patients with advanced dementia,^65-67^ or improves health outcomes in patients with poor nutritional status,^68^ some doctors feel that CANH is beneficial in the prevention of aspiration pneumonia, despite there being no robust evidence of this.^69-72^ Studies have shown that doctors who are more experienced in the care of dying patients are less likely to prescribe CANH^73^ and that doctors are more likely than nurses to discourage CANH in end-of-life care.^74^ A 2014 study of 53 492 hospitalizations of patients with advanced dementia found that general physicians were less likely than specialists to recommend CANH.^75^ Studies have also shown that the decision for or against CANH may be partly related to costs,^76,77^ staff availability,^17^ and the fear of litigation or negative publicity.^77,78^

The LACDP’s OCTGIR^4^ report highlighted the need to implement guidelines and improve training to deliver high-quality end-of-life care.

National Institute for Health and Care Excellence recommends that service providers are trained in the MCA Code of Practice,^79^ including consent, best interests decision-making, the role of Independent Mental Capacity Advocates, advanced care planning, ADRT, and lasting powers of attorney. They recommend training in assessing the hydration status of patients and discussing the risks and benefits of hydration with the patient and family.^80^ Royal College of Physicians advise that staff receive special training in administering food and fluids.^28^

## Discussion

I feel confident that the guidelines discussed are systematic, rigorous, and evidence based, with quality standards and overseen by a core group from the BMA, RCP, NICE, GMC, and LACDP along with a number of respected experts. However, it is important to be mindful that data from selected groups of patients, for example, patients dying from cancer, cannot be extrapolated to all dying patients. Continuous efforts should be made to expand the evidence base on which these guidelines are formed.

As OCGTIR rightly highlights, individualized care plans regarding food and drink are paramount rather than rigidly sticking to guidelines. The LCP’s downfall was its tick-box uniformity, which did not allow enough consideration of varying diagnoses, physical and mental states, beliefs, and levels of capacity.

Certainly, CANH training needs to be improved. Either way, however, current guidelines are subjective rather than objective. Does this make the guidelines harder for health-care professionals to follow? Perhaps, but subjective is not the same as vague, and making an objective CANH policy would be an impossibility, given the uniqueness of every patient’s dying phase.

The key seems to be being prepared in as many domains as possible: considering the diagnosis and clinical state; discussing the pros and cons of CANH several times with patients and families; and addressing cultural and religious beliefs, hopes and fears, and physical and psychological symptoms. Then, any uncertainties regarding CANH will be easier to manage.

## Conclusion

In the final days and hours of life, the risks and burdens of CANH generally outweigh any potential clinical benefit, but where started or continued, regular reviews of clinical benefit are needed. However, the patient’s wishes should be considered, ideally previously detailed in an advanced care plan. Sensitive and recurrent discussions are needed with the patient or family, seeking the patient’s best interests with a kind, flexible, and individualized approach.

## Supplemental Material

Supplementary_Material_xyz30949f811779a - To What Extent Does Clinically Assisted Nutrition and Hydration Have a Role in the Care of Dying People? Click here for additional data file. Supplementary_Material_xyz30949f811779a for To What Extent Does Clinically Assisted Nutrition and Hydration Have a Role in the Care of Dying People? by Adam Nicholas Carter in Journal of Palliative Care
