# Supplementary material for: To What Extent Does Clinically Assisted Nutrition and Hydration Have a Role in the Care of Dying People?
Source: J Palliat Care. 2020 Mar 4;35(4):209–16. doi: 10.1177/0825859720907426 (PMC7506871; doi:10.1177/0825859720907426)
Supplement: Supplementary_Material_xyz30949f811779a - To What Extent Does Clinically Assisted Nutrition and Hydration Have a Role in the Care of Dying People? [file Supplementary_Material_xyz30949f811779a.pdf]

# Supplementary Material

## Figure S1: The Principles of the Mental Capacity Act 2005

**The Principles of the Mental Capacity Act 2005**

1. A person must be assumed to have capacity unless it is established that he lacks capacity.
2. A person is not to be treated as unable to make a decision unless all practicable steps to help him to do so have been taken without success.
3. A person is not to be treated as unable to make a decision merely because he makes an unwise decision.
4. An act done, or decision made, under this Act for or on behalf of a person who lacks capacity must be done, or made, in his best interests.
5. Before the act is done, or the decision is made, regard must be had to whether the purpose for which it is needed can be as effectively achieved in a way that is less restrictive of the person's rights and freedom of action.

***Figure S1: The Principles of the Mental Capacity Act 2005***
Adapted from: Mental Capacity Act 2005. https://www.legislation.gov.uk/ukpga/2005/9/contents. Accessed January 2, 2019.

**Figure S2: The two-stage test to assess capacity under the Mental Capacity Act 2005**

**The two-stage test to assess capacity under the Mental Capacity Act 2005**

In order to decide whether an individual has the capacity to make a particular decision you must answer two questions:

**Stage 1**. Is there an impairment of or disturbance in the functioning of a person's mind or brain? If so,
**Stage 2**. Is the impairment or disturbance sufficient that the person lacks the capacity to make a particular decision?

The MCA says that a person is unable to make their own decision if they cannot do one or more of the following four things:

- understand information given to them
- retain that information long enough to be able to make the decision
- weigh up the information available to make the decision
- communicate their decision – this could be by talking, using sign language or even simple muscle movements such as blinking an eye or squeezing a hand.

Every effort should be made to find ways of communicating with someone before deciding that they lack capacity to make a decision based solely on their inability to communicate. Also, you will need to involve family, friends, carers or other professionals.

The assessment must be made on the balance of probabilities – is it more likely than not that the person lacks capacity? You should be able to show in your records why you have come to your conclusion that capacity is lacking for the particular decision.

***Figure S2: The two-stage test to assess capacity under the Mental Capacity Act 2005***
Adapted from: Social Care Institute for Excellence. Mental Capacity Act 2005 at a glance. https://www.scie.org.uk/mca/introduction/mental-capacity-act-2005-at-a-glance#assessment. Published 2009. Accessed January 2, 2019.
